# Autoantibodies in breast cancer sera are not epiphenomena and may participate in carcinogenesis

**DOI:** 10.1186/s12885-015-1385-8

**Published:** 2015-05-15

**Authors:** Félix Fernández Madrid, Marie-Claire Maroun, Ofelia A Olivero, Michael Long, Azadeh Stark, Lawrence I Grossman, Walter Binder, Jingsheng Dong, Matthew Burke, S David Nathanson, Richard Zarbo, Dhananjay Chitale, Rocío Zeballos-Chávez, Carol Peebles

**Affiliations:** 1 Department of Internal Medicine- Division of Rheumatology, Wayne State University School of Medicine, 640 Canfield, Detroit, MI 48201 USA; 2 Karmanos Cancer Institute, 4100 John R Street, Detroit, MI 48201 USA; 3 National Cancer Institute, NIH, Laboratory of Cancer Biology and Genetics, 37 Convent Dr. MSC 4255, Bldg 37 Rm 4032, Bethesda, MD 20892-4255 USA; 4 Department of Pathology, Children’s Hospital of Michigan, Wayne State School of Medicine, Detroit, MI 48201 USA; 5 Department of Pathology, Henry Ford Health System, Detroit, MI 48202 USA; 6 Center for Clinical Epidemiology & Biostatistics, School of Medicine, University of Pennsylvania, Philadelphia, PA USA; 7 Center for Molecular Medicine and Genetics, Wayne State University School of Medicine, 540 East Canfield, Detroit, MI 48201 USA; 8 INOVA Diagnostics Inc, 9900 Old Grove Rd, San Diego, CA 92131 USA; 9 Department of Diagnostic Radiology, Henry Ford Hospital and Health Network, 2799 West Grand Blvd, Detroit, MI 48202 USA; 10 Department of Surgery, Henry Ford Health System, 2799 West Grand Boulevard, Detroit, MI 48202 USA; 11 Department of Pathology and Laboratory Medicine, Henry Ford Hospital, 2799 West Grand Blvd, Detroit, MI 48202 USA; 12 Department of Pediatrics, Children’s Hospital of Michigan, Wayne State School of Medicine, Detroit, MI USA

**Keywords:** Autoantibodies, Autoimmunity, Immunogenicity, Breast cancer, Carcinogenesis, Centrosomes, Mitochondria, Centromeres, Cytoskeleton

## Abstract

**Background:**

The objective of this work was to demonstrate that autoantibodies in breast cancer sera are not epiphenomena, and exhibit unique immunologic features resembling the rheumatic autoimmune diseases.

**Methods:**

We performed a comprehensive study of autoantibodies on a collection of sera from women with breast cancer or benign breast disease, undergoing annual screening mammography. All women in this study had suspicious mammography assessment and underwent a breast biopsy. We used indirect immunofluorescence, the crithidia assay for anti-dsDNA antibodies, and multiple ELISAs for extractable nuclear antigens.

**Results:**

Autoantibodies were detected in virtually all patients with breast cancer, predominantly of the IgG1 and IgG3 isotypes. The profile detected in breast cancer sera showed distinctive features, such as antibodies targeting mitochondria, centrosomes, centromeres, nucleoli, cytoskeleton, and multiple nuclear dots. The majority of sera showing anti-mitochondrial antibodies did not react with the M2 component of pyruvate dehydrogenase, characteristic of primary biliary cirrhosis. Anti-centromere antibodies were mainly anti-CENP-B. ELISAs for extractable nuclear antigens and the assays for dsDNA were negative.

**Conclusions:**

The distinctive autoantibody profile detected in BC sera is the expression of tumor immunogenicity. Although some of these features resemble those in the rheumatic autoimmune diseases and primary biliary cirrhosis, the data suggest the involvement of an entirely different set of epithelial antigens in breast cancer. High titer autoantibodies targeting centrosomes, centromeres, and mitochondria were detected in a small group of healthy women with suspicious mammography assessment and no cancer by biopsy; this suggests that the process triggering autoantibody formation starts in the pre-malignant phase and that future studies using validated autoantibody panels may allow detection of breast cancer risk in asymptomatic women.

Autoantibodies developing in breast cancer are not epiphenomena, but likely reflect an antigen-driven autoimmune response triggered by epitopes developing in the mammary gland during breast carcinogenesis. Our results support the validity of the multiple studies reporting association of autoantibodies with breast cancer. Results further suggest significant promise for the development of panels of breast cancer-specific, premalignant-phase autoantibodies, as well as studies on the autoantibody response to tumor associated antigens in the pathogenesis of cancer.

**Electronic supplementary material:**

The online version of this article (doi:10.1186/s12885-015-1385-8) contains supplementary material, which is available to authorized users.

## Background

Breast Cancer [BC] is a major public health problem and the most frequent cause of death in women throughout the world. It has been estimated that BC is responsible for nearly 14% of all female cancer deaths [1], and there has been recent renewed interest in the regulation of cancer development by the immune system. It is generally agreed that cancer immunoediting is responsible for both eliminating tumors and sculpting the immunogenic phenotype of tumors [2]. This process is thought to be mediated by immune cells and no role is considered for the autoantibody response to tumor associated antigens [TAAs]. In this context, autoantibodies detected in sera from BC and other solid tumors have been shown to recognize multiple TAAs [3-10]. The participation of B cells in a Th2 polarized response has recently been paradoxically associated with BC progression [11], but the mechanism by which B cell activation may promote BC progression is unclear. Our studies on autoantibodies in malignancies strongly suggested that cancer sera exhibit immunologic features that are common in the rheumatic autoimmune diseases [ADs]. We have shown that anti-collagen antibodies [12] and antinuclear antibodies [ANA] [5,13] are found in the sera from lung cancer and head and neck cancer patients as frequently as in the systemic ADs such as rheumatoid arthritis [RA] and systemic lupus erythematosus [SLE]. With the use of molecular techniques and high throughput analyses, we and others have clearly shown that autoantibody classifiers have been constructed with high sensitivity and specificity for the diagnosis of breast and other cancers [7-10]. It is well known that autoantibodies (such ANAs in SLE and rheumatoid factors and anti-cyclic citrullinated peptides antibodies in RA) can be detected many years before the onset of these ADs [14,15]. We and others have also found autoantibodies in the sera of patients with cancer before clinical diagnosis [3,4,6], notably suggesting that the breakdown of tolerance to tumor antigens is an early event in carcinogenesis. The diagnostic value of autoantibodies as immune biomarkers in the systemic and organ-specific ADs such as SLE, RA, scleroderma [16-20], and primary biliary cirrhosis [PBC] [21-23] is well established. Despite the many reports on autoantibodies determined by immunofluorescence [IFA] in cancer sera, their significance remains unclear [24]. Although ANAs have been known to occur in BC sera for several decades [25], a comprehensive study of autoantibodies on a large collection of sera from patients with pathology-proven BC and a real-life control group using classical techniques has not been reported. The objective of this work was to demonstrate that the autoantibodies detected in BC sera have unique immunological features, resembling the model epitomized by the rheumatic and organ-specific ADs [16-20]. In this study, detection of autoantibodies in the sera from practically all women with BC provides compelling evidence that an antigen-driven autoantibody response takes place in BC. Moreover, we report here that the autoantibody profile detected in BC sera has distinctive features, probably reflecting unique BC-associated antibody specificities targeting antigens in the mitochondria, the centrosomes, the spindle apparatus, the nucleoli, and the cytoskeleton.

## Methods

We used IFA on HEp-2 cells [20] to perform a comprehensive survey of autoantibodies in sera from women undergoing annual screening mammography with suspicious assessment [26]. We elected to use IFA on HEp-2 cells as the objective of our study was to demonstrate that the autoantibodies detected in BC sera have unique immunological features resembling the model epitomized by the rheumatic and organ-specific ADs. IFA on HEp-2 cells is recognized as the present standard to determine ANAs in the clinical laboratory; it is also a time-honored method used in all the classic autoantibody studies in the rheumatic ADs [16-20] and in the many reports of ANAs in cancer sera following Wasserman’s report in 1975 [25]. Though HEp-2 cells were originally derived from a patient with laryngeal carcinoma, multiple studies have shown these cells to provide an ideal standardized substrate for IFA that lends itself to comparison studies of immune reactivity in many diverse diseases including cancer and the rheumatic ADs [16,20]. Since anti-mitochondrial antibodies [AMA] were detected by IFA (among other reactivities), the mitochondrial specificity of these autoantibodies was confirmed by IFA staining of stomach and kidney mitochondria from rodent sections. These substrates are very rich in mitochondria and are typically used to validate mitochondrial reactivity found on HEp-2 cells [21]. We also performed immunoblots [IBs] of BC proteins [27], crithidia luciliae assay for anti-dsDNA antibodies [28], and multiple ELISAs for extractable nuclear antigens [ENAs], anti-centromere antibodies [CENPs], NSP1 antibodies and for the M2 component of pyruvate dehydrogenase.

### Patient material

Cases of ductal carcinoma in situ [DCIS] and invasive ductal carcinoma [IDC] of the breast studied in this work, as well as controls with benign breast disease [BBD], were obtained from a population of women ≥ 40 years old undergoing annual screening mammography at Henry Ford Health System [HFHS]. Written informed consent was obtained from each woman participating in the study. These women had BI-RADS4 mammography assessment [26]. BI-RADS is a quality assurance tool designed to standardize mammography reporting for radiologists with sufficient concern to urge performance of a breast biopsy [26]. Since approximately 20% of women with suspicious mammography are usually found to have BC and about 80% have BBD, additional sera from cases of DCIS and IDC of the breast were also obtained from the Tissue Procuring Facility of HFHS. These sera were previously collected and stored frozen until use from women who had breast cancer after obtaining a written informed consent for the archived sera to be used for research purposes. Pathologic diagnoses of cases were made by breast biopsy performed at the time of mammography and prior to treatment. We used one hundred sera from women with DCIS, one hundred with IDC, and one hundred with BBD as the main control [Table 1]. Demographics and data from pathology and mammography reports were obtained from the electronic database of HFHS. Sera from a group of healthy hospital female nurses [N = 100] and female patients with the diagnosis of osteoarthritis [N = 122] recruited from the rheumatology clinic at Wayne State University were also used in this study as additional controls. Written informed consents were obtained from all the additional controls. Cases and controls with past or present history of SLE, RA, scleroderma, or any other rheumatic ADs were excluded. This study was approved by the IRBs at HFHS and Wayne State University.

**Table 1:** **IFA and IBs in BC and control sera**

	Convenience control female	Osteoarthritis control female	Benign breast disease	Ductal carcinoma in situ	Invasive carcinoma
**Age in years mean [range]**	53 [40–62]	63 [40–87]	54.7 [34–85]	58.9 [32–87]	60.7 [31–88]
**Low titer ≥ 1:100 [%]**	15	32	61	70	76
**High titer ≥ 1:320–640 [%]**	2	6	27	46	51
**Immunoblots ≥1:500 [%]**	ND	ND	39	85	85
**IFA + immunoblots [%]**	ND	ND	66	95	99

All reactive sera at 1:100 by IFA were titrated to the end point. ND, not done.

### Immunoblots of breast cancer proteins

Immunoblots of BC proteins were probed with sera from cases and BBD controls at a serum dilution of 1:500. Protein extracts were prepared by the method of Wood and Earnshaw [29] from eight established BC cell lines, MCF-7, DCIS.com, SKBR, T47D, SUM44, SUM102, SUM149, and SUM159, which were gifts from Drs. Frederick Miller and Stephen Ethier. These different breast cancer cell lines were used to produce the protein extracts for the IBs to account for the heterogeneity of BC. The pooled extracts from the 8 cell lines were separated by SDS-PAGE and transferred to nitrocellulose [27]. The IBs were developed with secondary antibodies for IgG1-4, IgA, and IgM [Sygnus Technologies, Southport, North Carolina, USA]. Molecular mass standards [Sigma Chemicals, USA] were used to determine the molecular mass of proteins recognized by IgG on IBs.

### Immunofluorescence techniques

HEp-2 cells [American Type Culture Collection from human laryngeal carcinoma] and fluorescent anti-IgG conjugates [INOVA, San Diego, California, USA] were employed to detect ANAs and anti-cytoplasmic antibodies [20] using BC and control sera initially diluted to 1:100. For IFA staining, slides were reacted for 30 min with the initial dilution of sera from cases and controls at room temperature to assure moist conditions. After rinsing and washing in PBS for 5 min, slides were reacted with high sensitivity IgG conjugate [INOVA] for 30 min followed again by washing in PBS for 5 min and covering with a cover slip. Nuclear or cytoplasmic reactivities equal or greater than 1:100 were considered positive and all reactive sera were titrated to the end point. Sera reacting at 1:100 to 1:160 dilution were considered to have low titer ANAs, and those reactive at ≥1: 320–640 dilution were considered to be high titer [Table 1]. Nuclear and cytoplasmic fluorescence including homogeneous, fine and coarse speckled, anti-centromere, and AMA patterns as well as centrosome/spindle apparatus, multiple nuclear dots [MNDs], and cytoskeletal fluorescence were read by three independent observers [CP, FFM, and ML] who were in agreement in more than 97%. These immunofluorescence patterns are well established features of antinuclear and anti-cytoplasmic antibodies reported in hundreds of publications over several decades [18-20,23].

### Determination of anti-dsDNA and specific ELISAs for ENA and other autoantibodies

To determine whether BC and control sera with positive ANAs had anti-dsDNA antibodies, we used the crithidia luciliae assay [28] to test all sera from the three groups exhibiting a homogeneous pattern with titers of ≥1: 320–640. All AMAs detected by IFA on HEp-2 cells were verified by fluorescence staining of rodent stomach and renal tubuli mitochondria [Ortho Diagnostic, Raritan, New Jersey, USA] [21] at a dilution of 1:100. All sera showing positive ANAs were tested by ELISA [INOVA, San Diego, California, USA] at a dilution of 1:160 for the presence of ENAs [17,19].

All sera from cases and controls showing AMAs by IFA and an equal number of ANA positive and AMA negative sera were tested for the M2 antigen complex of pyruvate dehydrogenase by ELISA at a 1:100 dilution. ELISA was performed as a solid phase enzyme labeled immunosorbent assay in microwells coated with purified mitochondrial antigen [Orgentec Diagnostika, Mainz, Germany]. Controls, calibrators, and patient sera were incubated in the microwells. Unbound antibody and other serum proteins were removed by washing. Bound antibodies were incubated with an enzyme labeled anti-human IgG conjugate and unbound conjugate was removed by washing. Specific enzyme substrate [pNPP] was added and antibodies were detected colorimetrically. Identical aliquots of AMA-positive and AMA-negative sera detected by IFA were tested by ELISA using the r-PDCE2 antigen in the laboratory of Dr. Eric Gershwin at UC Davis California, USA at a 1:250 dilution. ELISA was also used to evaluate BC and BBD sera for antibodies to centromere proteins [CENP] A and B in all ANA positive sera. Recombinant centromere proteins A or B were bound to the microwells and incubated with patients’ sera, controls, and calibrators. After washing to remove unbound antibodies and other serum proteins, horseradish peroxidase conjugated anti-human IgG was used to detect bound antibodies forming a conjugate/antibody/antigen complex. After washing to remove unbound conjugate, specific enzyme substrate was added to the wells and antibodies were detected as before. Using a similar technique, all sera showing AMAs and/or MNDs by IFA were tested for NSP1 [Orgentec Diagnostika, Mainz, Germany].

## Results

### The prevalence of autoantibodies in BC and BBD control sera was high

The combined use of IFA on HEp-2 cells and IBs of BC proteins detected autoantibodies in virtually all sera from patients with BC [Table 1]. IFA of BC and control sera revealed a spectrum of autoantibodies with maximal reactivity in sera from patients with IDC and decreasing reactivity in DCIS and BBD sera; minimal nuclear or cytoplasmic reactivity was shown in the sera from patients with OA and from healthy hospital nurses [Table 1]. The prevalence of autoantibodies in BC sera was well within the range reported in the rheumatic ADs [16,20]. High titer autoantibodies were most common in sera from patients with IDC and DCIS, and less frequent in control BBD sera. The ANA reactivities in both the convenience group and the group of females with OA were within the range reported in previous studies [20,24,30-32]. The finding of high titer antibodies in some healthy women in the BBD control group is of interest because high titer antibodies [>1:320–640] are seldom found in sera from healthy individuals [20,30-32]. In this respect, it is relevant that the BBD group was not a convenience control, since the healthy women in this control group were undergoing annual screening mammography and had suspicious mammography findings [26]. The autoantibodies detected by IBs in BC and BBD sera were predominantly of the IgG1 and IgG3 subclasses; less frequently detected were IgG2, IgG4, IgM, or IgA [data not shown].

### IFA of BC sera revealed a distinct autoantibody profile

**Figure 1:**
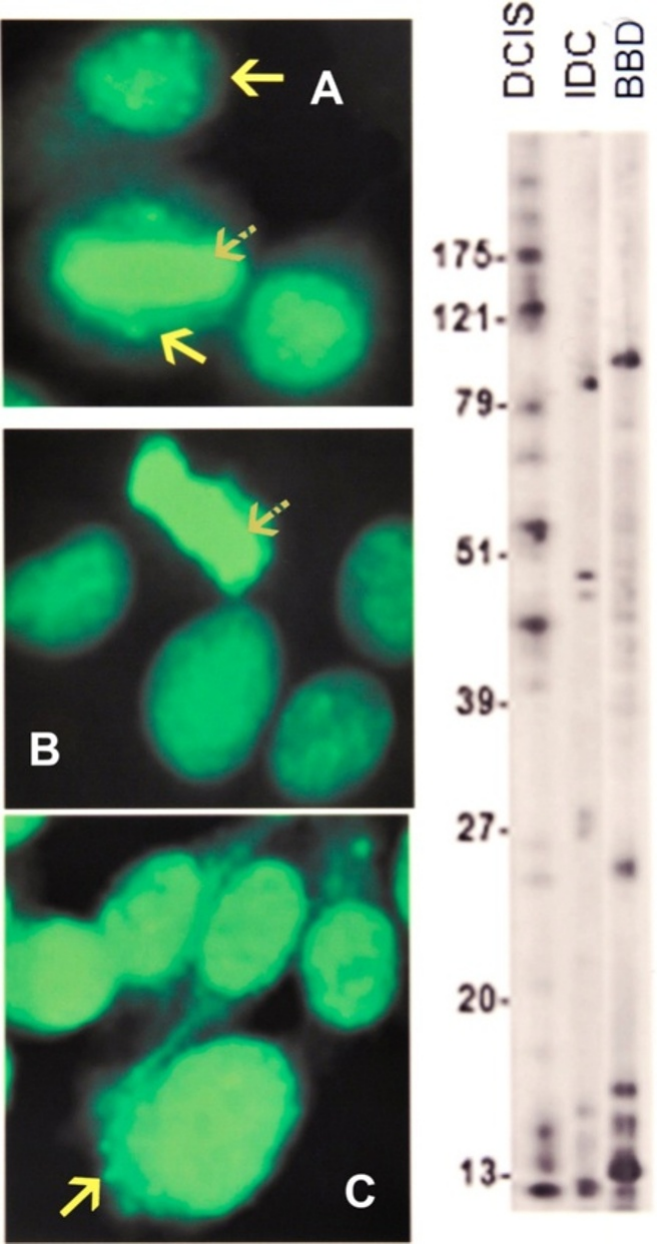
In addition to the homogeneous pattern in all three specimens, the serum in **A** from a patient with DCIS had a mixed pattern with the lower solid arrow depicting anti-centrosome antibodies while an upper solid arrow shows MNDs. **B** corresponds to a serum from a patient with IDC. A striped arrow in **A** and **B** show a positive metaphase plate as seen in the homogeneous pattern. The arrow in **C** points to mitochondrial fluorescence in a specimen from a healthy woman with BBD. The immunoblots done at a 1:500 dilution in all figures, showed the presence of many more IgG antibodies than those recognized by IFA.

**Figure 2:**
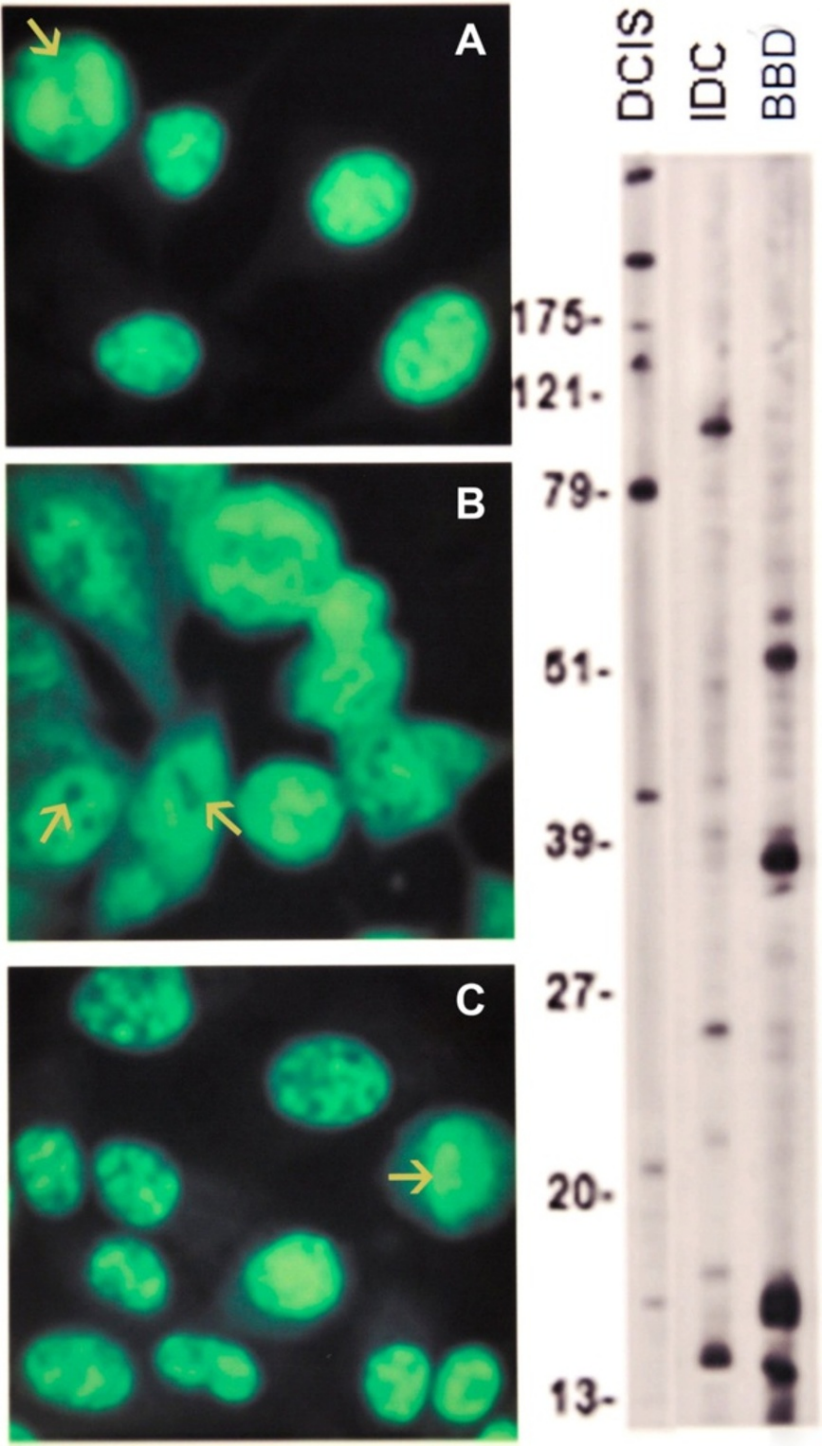
The three specimens depicted in **A**, DCIS, **B**, IDC, and **C**, BBD show a speckled pattern. The arrow in **A** shows a negative metaphase plate; the arrows in B show the negative unstained images of nucleoli while the arrow in **C** shows a positive metaphase plate suggesting a mixed homogeneous and speckled pattern.

**Figure 3:**
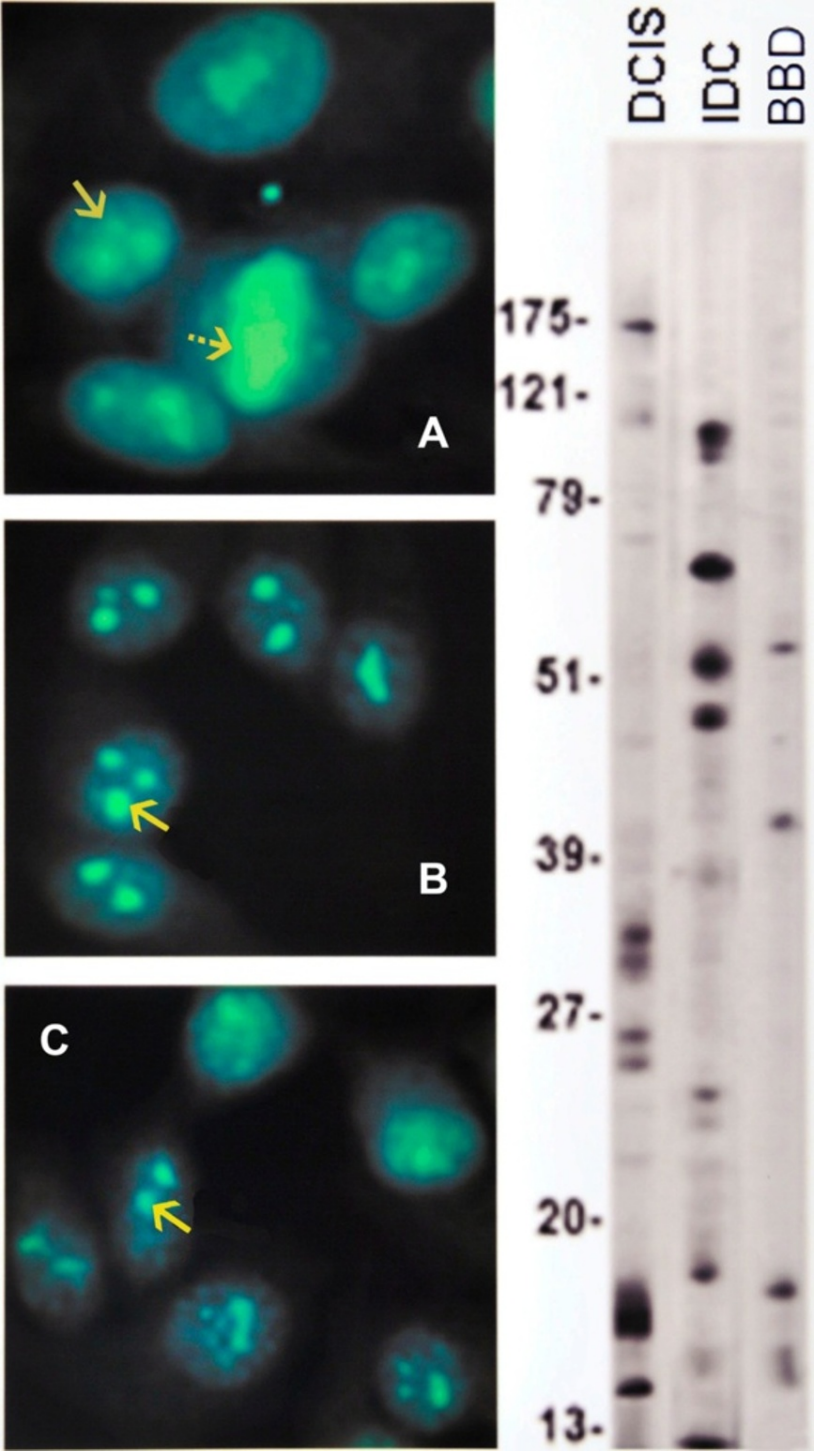
The nucleolar pattern is shown in **A**, DCIS, **B**, IDC, and **C**, BBD, indicated by solid arrows. An additional striped arrow in **A** points to a positive metaphase plate indicating a concomitant homogeneous pattern.

**Figure 4:**
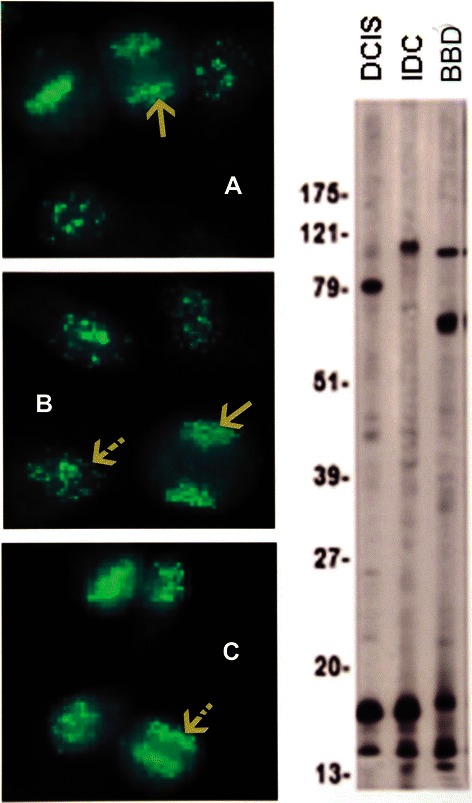
Anti-centromere antibodies are shown in **A**, DCIS, **B**, IDC, and **C**, BBD. The solid arrows in **A** and **B** indicate the fluorescent chromosomes aligned in telophase; the striped arrow in **B** points to a resting interphase cell, while the arrow in **C** shows the fluorescent centromeres aligned in metaphase. The immunoblots show the presence of an 80 kDa IgG band corresponding to CENP-B in a DCIS serum.

IFA showed a diversity of autoantibody patterns with homogeneous, speckled, nucleolar, and centromere fluorescence; these patterns are classically recognized in SLE, RA, and scleroderma [20] [Table 2 and Figures 1, 2, 3 and 4]. Although these patterns are identical to those seen in the rheumatic ADs, the autoantibody profile detected in BC sera had distinctive features. The homogeneous pattern was predominant in women with DCIS and BBD, and least common in women with IDC [Table 2 and Figure 1]. In this study all sera with high titer ANAs and homogeneous pattern were non-reactive in the crithidia assay for anti-dsDNA [data not shown]. Fine and coarse speckled patterns were most frequent in IDC sera and their frequency decreased in the sera from women with DCIS and BBD [Table 2 and Figure 2]. The speckled pattern in the rheumatic ADs is often the expression of antibodies to a group of proteins known as ENAs [17-19]. In contrast, all sera from BC cases and controls displaying coarse or fine speckled pattern were negative for Sm, RNP, SS-A[Ro], SS-B[La], Scl-70, and Jo-1 antibodies by ELISA [data not shown]. Nucleolar fluorescence was very frequent in BC and less prominent in BBD sera [Table 2 and Figure 3]. Anti-centromere antibodies were also found in all three groups [Table 2 and Figure 4]. Except for three sera in which there was insufficient material, all sera showing anti-centromere antibodies by IFA had anti-CENP-B by ELISA [data not shown].

**Table 2:** **ANAs and anti-cytoplasmic antibodies on HEp-2 cells in BC and non-cancer control sera**

IFA pattern [%]*	Convenience control female	Osteoarthritis control female	Benign breast disease	Ductal carcinoma in situ	Invasive carcinoma
**Homogeneous**	6	7	46	41	29
**Speckled**	7	11	8	17	38
**Anti-nucleolar**	2	3	30	27	32
**Anti-centromere**	0	0	2	5	5
**Anti-centrosome**	0	0	16	18	12**
**Anti-mitochondrial**	0	0	10	11	22
**Multiple nuclear dots**	0	0	3	3	5
**Cytoskeletal**	0	0	0	3	3
**Mixed pattern**	2	9	37	45	51
**Other**	0	1	3	5	4

*Percent of individual patterns do not add up to 100% due to high frequency of mixed patterns.

**Percent likely underestimates prevalence of anti-centrosome antibodies because of the masking effect of the high frequency of cytoplasmic speckles and heavy mitochondrial fluorescence in sera from invasive breast carcinoma.

### The AMAs detected in breast cancer and in primary biliary cirrhosis sera target different mitochondrial antigens

**Figure 5:**
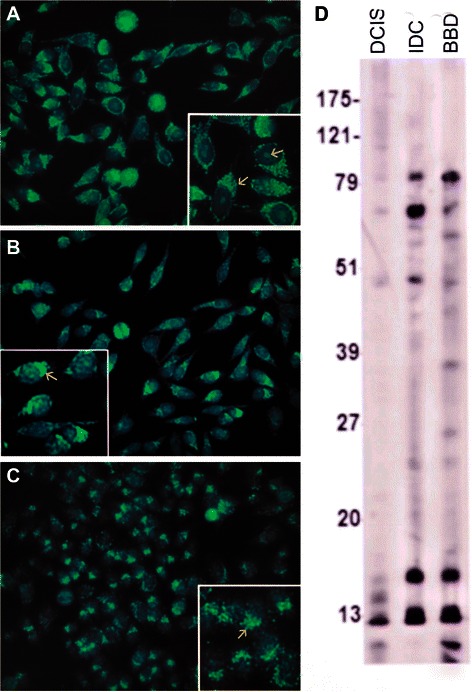
AMAs in sera from BC cases and healthy women are shown in **A**, DCIS, **B**, I DC and **C**, BBD. Lower arrow in **A**, [inset] points to massive mitochondrial fluorescence while upper arrow shows a nucleolus. The arrows in **B** and **C** [insets], point to the cytoplasm studded with mitochondria. **D**, Immunoblot of BC proteins probed with DCIS, DCIS, and BBD sera.

**Figure 6:**
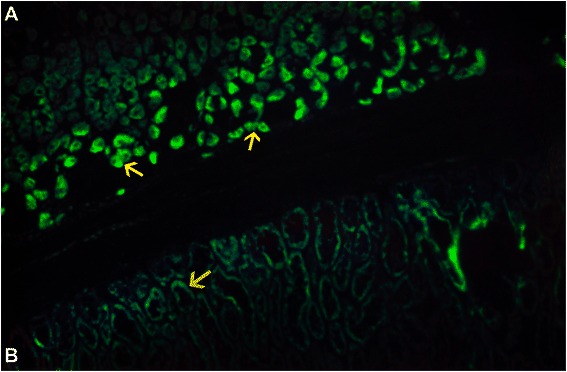
AMAs in BC and BBD sera decorate mitochondria in rodent stomach parietal cells [upper arrow] and renal tubuli [lower arrows].

**Figure 7:**
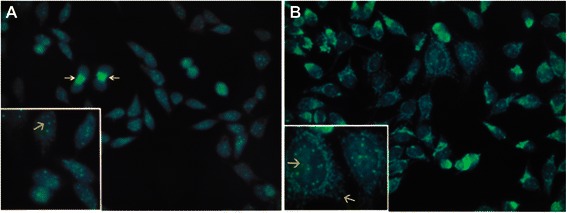
Mixed patterns with BC sera with multiple nuclear dots. **A**. DCIS. MNDs [arrow in inset] and midbody fluorescence [upper arrow]; **B**. IDC. Mixed pattern with MNDs [upper arrow] and AMAs [lower arrow].

The predominance of AMAs in BC detected by IFA is a distinctive feature that may be characteristic of autommunity in BC, and was consistently found in all three groups [Table 2 and Figure 5 A, B, C, D]. *AMAs at high titer as found in BBD control sera are not found in healthy women* [23,30-32]. The presence of AMAs in BC and BBD sera was confirmed on rodent kidney and stomach sections showing the characteristic mitochondrial fluorescence in renal tubuli and stomach parietal cells [Figure 6]. The AMAs in BC sera were indistinguishable from the AMAs detected by IFA in PBC. Consequently, we tested all AMA positive BC sera on ELISA for the M2 antigen complex characteristic of PBC, which is known to correspond to the 2-oxo-acid dehydrogenase complex [22] [Additional file 1]. ELISA showed unequivocal reactivity with the M2 antigen complex in only one serum from a patient with IDC. This serum also showed multiple nuclear dots [MNDs], a combination which is thought to be characteristic of PBC [21,23,33]. The ELISA results on the M2 antigen were confirmed in Dr. Eric Gershwin’s laboratory [data not shown]. MNDs fluorescence is characterized by the staining of a variable number, 3 to 30 dots distributed over the nucleus, sparing the nucleoli, and not staining the chromosomes during mitosis [33]. Mixed patterns involving the association of AMAs and MNDs were detected in IDC and DCIS sera [Figure 7] as well as in some BBD control sera [data not shown]. Since the single BC patient with antibody to the M2 antigen complex could coincidentally have PBC, we retrieved clinical data and liver function tests on all patients whose sera showed AMAs by IFA. During a 10-year follow-up none of these patients had a diagnosis of PBC, developed liver disease such as autoimmune hepatitis, or had abnormal liver function tests that could be attributed to PBC. With the possible exception of one patient with IDC, PBC was excluded as an explanation of mitochondrial reactivity as the majority of the BC sera did not react with the M2 pyruvate dehydrogenase antigen complex. It is clear, therefore, that the AMAs detected by IFA reflect different mitochondrial specificities. In contrast with PBC in which MNDs are frequently associated with NSP1 reactivity [33], ELISAs performed in all BC and control sera with MNDs were negative for NSP1 [data not shown], suggesting that the MND fluorescence in BC sera may be related to reactivity to other antigens.

### Centrosomes, the spindle apparatus, and the cytoskeleton are targeted by autoantibodies in BC

**Figure 8:**
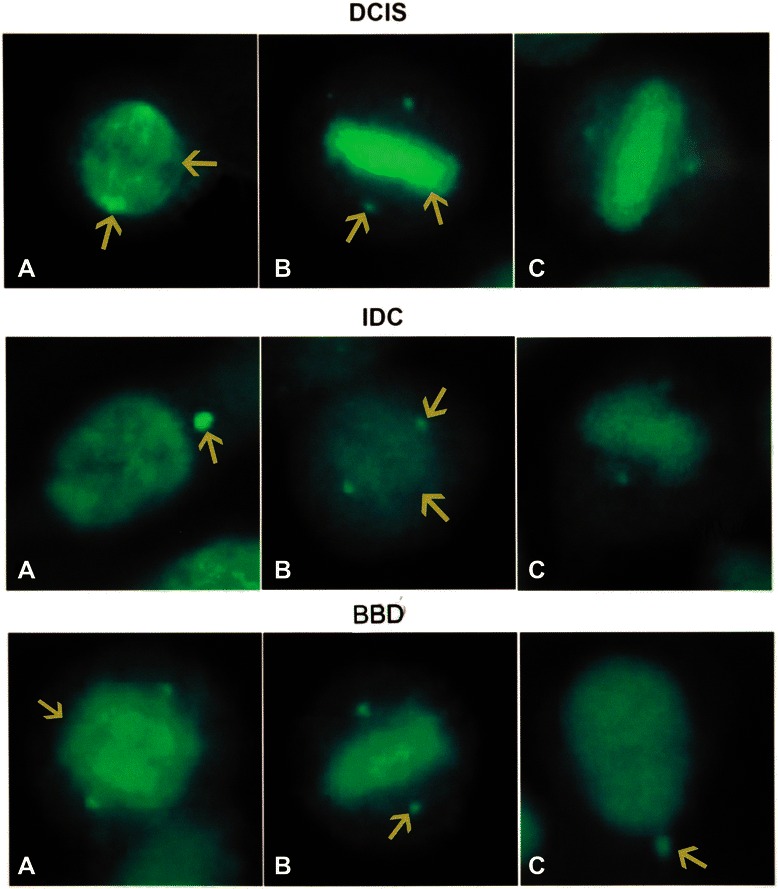
Anti-centrosome and anti-spindle antibodies. Upper row shows sera from DCIS, middle row sera from IDC and lower row from healthy women with BBD. In the upper row, the lower arrow in **A** indicates a centrosome fluorescence while the upper arrow points to the mitotic spindle; In the middle row the arrow in **A** depicts a centrosome while the upper arrow in **B** points to the centrosome and the lower arrow to the mitotic spindle; In the lower row, the arrow in **A** signals a mitotic spindle while the arrows in **B** and **C** show centrosome fluorescence.

**Figure 9:**
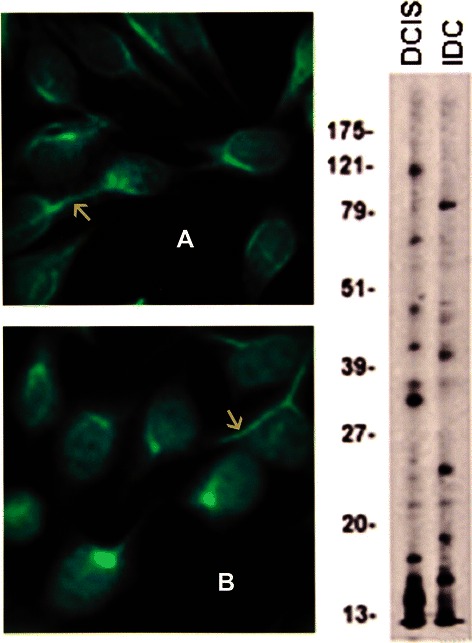
Anti-cytoskeletal antibodies **A**, DCIS, **B**, IDC. **A** and **B** show fluorescence decorating cytoskeletal antigens. Only occasional cytoskeletal antibodies were found in the sera from healthy control women with BBD.

The abundance of antibodies to centrosomes and spindle apparatus in our samples was notable. Anti-centrosome antibodies were frequently present in IDC, DCIS, and BBD sera [Table 2 and Figure 8]. These antibodies are found in a substantial proportion of sera from patients with BC as well as from healthy women. The prevalence of anti-centrosome antibodies may be even higher, however, as quantification of antibodies decorating the centrosomes can be masked in the presence of mixed patterns including speckled, mitochondrial, cytoskeletal, and other cytoplasmic fluorescence interfering with their detection. Frequency of autoantibodies decorating filamentous structures was low but consistent in BC, indicating reactivity with cytoskeletal antigens; this was not found in BBD sera [Table 2 and Figure 9]. Antibodies to centromeres, centrosomes, mitochondria, MNDs, or cytoskeletal antigens were not detected in either the convenience group or in the sera from women with OA [Table 2].

## Discussion

ANAs have been known to be present in BC sera for several decades [25] but their significance remains unknown [24]. This is likely because autoantibodies are part of the normal immune response, and sera from healthy subjects exhibit a plethora of autoantibodies not related to cancer [30-32]. The application of genomics and proteomics to biomarker discovery allowed the identification of multiple autoantibodies in BC sera recognizing TAAs [3-10]. These studies strongly suggested the possibility that autoantibodies in cancer sera were potentially useful biomarkers for the early diagnosis of BC. The seminal work establishing the role of autoantibodies as diagnostic biomarkers in the rheumatic ADs [16-20] suggested the hypothesis that the model epitomized by the rheumatic ADs is highly relevant to explain the plethora of autoantibodies detected in cancer sera. Importantly, PBC as an organ-specific autoimmune disease is characterized by a set of autoantibodies with mitochondrial specificity with recognized diagnostic value [21-23]. In this work we attempted to demonstrate that autoantibodies in BC sera have unique immunological features, as in those found in the rheumatic and some organ-specific ADs. We show here that autoantibodies reacting with antigens located in several important cell organelles (including mitochondria, centromeres, nucleoli, centrosomes, and the mitotic spindle) are consistently found in sera from women with suspicious mammography findings. The level of these autoantibodies was highest in women with IDC, lesser in women with DCIS, and still lower but above background levels in healthy women with BBD [Table 2]. Moreover, AMAs, anti-centromere, and anti-centrosome antibodies are not components of the autoantibody repertoire of normal healthy women [30-32]. These findings suggest that, in the future, the combination of suspicious mammography and autoantibody signatures could potentially identify a group of women in the early stages of breast carcinogenesis. Here we provide evidence that most of the antigens targeted by autoantibodies in BC sera differ from those involved in the rheumatic and organ-specific ADs. Sera from patients with SLE frequently exhibit a homogeneous pattern and anti-dsDNA or anti-histone antibodies [18,20]. Although anti-dsDNA antibodies were not found in sera from women with homogeneous pattern of nuclear fluorescence, we have detected anti-histone antibodies in BC sera by immunoscreening a cDNA library of BC proteins with BC sera [unpublished data, FFM et al.]. The speckled pattern is found in scleroderma and other rheumatic ADs [16,20] and very frequently in IDC of the breast. None of the BC sera exhibiting the speckled pattern [Table 2 and Figure 2] reacted with ENAs on ELISA as is frequently the case in the rheumatic ADs. Anti-centromere antibodies which are characteristic of limited scleroderma, or CREST syndrome [34], were found in the sera from cases of BC and BBD controls. Most of the anti-CENPs detected in BC sera in this work [Table 2 and Figure 4] were anti-CENP-B, which have been reported in BC sera [35] and are prevalent in the rheumatic ADs [16,20]. Centromere protein abnormalities reflected by the presence of CENP antibodies are clearly common in BC. The consistent finding of CENP antibodies in BBD sera is notable, since these antibodies are seldom found in healthy women [23,30-32].

### Autoantibodies reacting with centrosome antigens are common in women with BC and BBD

Autoimmune sera contain autoantibodies targeting epitopes found in a family of proteins located on centrosomes [36]. Centrosome aberrations have long been reported in invasive and pre-invasive cancer [37,38]. In our study, anti-centrosome antibodies were detected frequently in both BC and BBD sera [Table 2 and Figure 8], but these autoantibodies were not prominent features in previous studies in healthy subjects [30-32]. Abnormal centrosome amplification and supernumerary centrosomes, as well as abnormalities in centrosome number, size, and morphology, have been observed in nearly all human tumor types including BC [38]. Centrosome defects have been associated with genetic instability [37,38] but the role of the centrosome in tumorigenesis is yet to be defined. The significance of anti-centrosome antibodies and autoantibodies reacting with proteins in the spindle apparatus in cancer sera is unclear. The novel findings reported here suggest that autoantibodies in BC sera are promising probes to identify centrosome proteins likely to be implicated in both autoimmunity and cancer. Chromosomal aberrations are the hallmark of cancer and autoantibodies develop early in carcinogenesis. Thus, the possibility should be investigated that autoimmunity to centrosome and mitotic spindle proteins may be involved in inducing genetic instability.

### High titer AMAs are frequently found in BC sera

AMAs detected by IFA in sera from women with BC and BBD [Figure 5] are indistinguishable from the mitochondrial fluorescence typically detected in the sera from patients with PBC [21-23]. AMAs reacting with the M2 mitochondrial antigen complex are diagnostic markers for PBC [22]. One serum from a woman with IDC of the breast reacted with the M2 antigen complex, suggesting that most of the AMAs in BC sera have specificities other than those found in PBC. The cases and BBD controls in our cohort that displayed AMAs in their sera did not have liver disease. Although liver function was normal in the only BC patient whose serum reacted with the M2 antigen complex, it is possible that this result could be due to coincidental PBC developing in a patient with BC. AMAs, MNDs, and anti-centromere antibodies as seen in BC sera in our study are classically detected in PBC [21-23,33] [Table 2, Figures 4, 5A-D and 7B]. Another similarity between PBC and BC is that ANAs are found in both conditions. The resemblance between the AMAs in BC and PBC, however, is limited to the mitochondrial origin of the antigens since most of the AMA-positive sera in BC did not react with the M2 antigen complex. This is in agreement with our report of two mitochondrial proteins, peripheral benzodiazepine associated protein-1 [PRAX-1] [39] and diazepam binding inhibitor related protein [40] recognized as autoantigens by BC sera [9,41]. Furthermore, although AMAs predominate over ANAs in PBC [21,23], ANAs predominate over AMAs in BC sera. MNDs are commonly found in PBC and with less frequency in the sera from BC patients. While in PBC, MNDs are associated with the NSP1 antigen [33], the MNDs seen in BC sera [Table 2 and Figure 7B] do not seem to be related to NSP1 antibodies. Thus, the distinctive serologic findings (AMAs, MNDs, centrosome, and nucleolar staining) observed in BC sera appear to reflect a distinct autoantibody repertoire, suggesting that autoimmunity to TAA residing in breast tissue is a prominent feature in BC.

### The autoantibodies found in some healthy women with BBD may be generated during the pre-malignant phase

Although many normal subjects exhibit low titer ANAs in their sera, relatively few healthy individuals have positive ANA tests at high titers [20,30-32]. The results of our study are not strictly comparable to previous reports since all our cases and controls were women, and women are known both to have higher levels of autoreactivity and to develop more robust immune responses than men [42]; this may partly explain the higher levels of autoantibodies in our group of healthy women with BBD. Nevertheless, the results from IFA in the sera from women with suspicious mammography assessment were notable for two reasons: the findings of relatively high ANA titers [≥1:320–640, Table 1] with high frequency of mixed patterns [Table 2] in some women with BBD, and the detection of AMAs and antibodies to both centromeres and centrosomes in these sera [Table 2 and Figures 4C, 5C and D, and Figure 8, lower row]. These results indicate that a group of healthy women undergoing annual screening mammography and shown by breast biopsy not to have BC have serologic findings not commonly found in healthy women [30-32]. These data suggest the hypothesis that the sera from some women in the control group having BBD may contain signatures that could in the future help identifying women at high risk for BC.

### Are autoantibodies participants in breast carcinogenesis as well as being biomarkers?

Both rheumatic ADs and cancer develop after a considerable latent period [43,44]. The evidence for multiple cooperating events in the pathogenesis of the rheumatic ADs resembling those observed in multistep carcinogenesis has been noted [43]. Somatic mutations are considered to be major factors in the pathogenesis of both autoimmunity and carcinogenesis [43,44]. Our findings indicate that autoantibodies in BC sera have unique immunological features, as do rheumatic and organ-specific ADs; this suggests that autoantibodies in BC sera are not epiphenomena and that they may, as in the rheumatic ADs, be participants in the process of carcinogenesis. Until recently, the participation of B cell activation and autoantibodies in the anti-cancer immune response attracted little attention [2]. However, infiltrating lymphocytes including B and T cells have been well described in breast tumor tissue, suggesting an antigen-driven immune response triggered by TAAs [45-47]. A causal relationship between chronic inflammation and cancer has been established for many solid tumors [48]. Recent studies have proposed a possible role of antibody and/or cytokine-mediated effects of B cells on cancer cells, paradoxically potentiating disease progression [11]. Although the consistent presence of activated B cells in BC tumor tissue has been clearly demonstrated, the mechanism by which B cells may promote cancer progression has not been established. In view of our findings, investigation of a hypothesis in the context of an appropriate genetic background is warranted regarding tumor antigen-triggered autoimmunity, and how it may inflict epithelial damage in the breast as the target organ through promotion of chronic inflammation and cancer progression. Future research based on this study may clarify whether immunotherapy in the treatment of BC should attempt to stimulate or suppress immunogenicity of TAAs in the efforts to modulate the immune system.

## Conclusions

The autoantibody profile detected by IFA in BC sera has distinct features reflecting a unique autoantibody repertoire. To our knowledge this is the first report of AMAs detected by IFA on HEp-2 cells in a substantial subset of patients with BC, distinct from the AMAs characteristic of PBC. The consistent finding of anti-centrosome antibodies in BC sera is novel and supports the possibility that centrosome autoimmunity might be involved in cancer pathogenesis. Detection of AMAs, anti-centromere, anti-centrosome antibodies, and MNDs in a fraction of women with suspicious mammography findings and BBD indicates that the process triggering autoantibody formation starts in the pre-malignant phase. Our findings suggest the hypothesis that autoimmunity triggered by TAAs may inflict epithelial damage to the breast, promoting chronic inflammation and cancer progression; i.e., that BC may behave as an organ-specific AD triggered by multiple epithelial and other breast antigens. Moreover, the reports of enhanced expression of some mitochondrial antigens on epithelial cells from aggressive phenotype BC [49], in conjunction with the known association of mitochondrial dysfunction [50] with aggressive forms of cancer, suggest that AMAs may be potential biomarkers of aggressive BC and that the investigation of the specificity of the AMAs found in BC sera may be rewarding.

## Additional file

Additional file 1:
**ELISA for determination of antibody reactivity with the M2 component of pyruvate dehydrogenase as described in methods.**

## Abbreviations

AMAs: Anti-mitochondrial antibodies
ANAs: Antinuclear antibodies
ADs: Autoimmune diseases
BBD: Benign breast disease
BC: Breast Cancer
CENPs: Centromere Proteins
DCIS: Ductal carcinoma in situ
ENA: Extractable nuclear antigens
HFHS: Henry Ford Health System
IBs: Immunoblots
IFA: Immunofluorescence assay
IDC: Infiltrating ductal carcinoma
mtDNA: Mitochondrial DNA
MNDs: Multiple nuclear dots
PBC: Primary biliary cirrhosis
RA: Rheumatoid arthritis
SLE: Systemic lupus erythemathosus
TAAs: Tumor associated antigens
